# From bench to bedside: experimental pharmacology approaches to overcome bioavailability, stability, and translational barriers of bioactive peptides from natural sources

**DOI:** 10.3389/fphar.2026.1863016

**Published:** 2026-08-12

**Authors:** Mamoudou Hamadou, Saurav Kumar Mishra, Abdulhalim Musa Abubakar, Bahri Başaran, Mune Mune Martin Alain

**Affiliations:** 1 Department of Biological Sciences, Biochemistry, Bioinformatics, and Bioactive Compounds for Health Promotion, Research Unit, Faculty of Science, University of Maroua, Maroua, Cameroon; 2 Department of Chemistry, COMSATS University, Islamabad, Pakistan; 3 Department Basic Science Education, National Advanced School of Engineering, University of Maroua, Maroua, Cameroon; 4 Department of Bioinformatics, University of North Bengal, Darjeeling, West Bengal, India; 5 Department of Chemical Engineering, Faculty of Engineering, Modibbo Adama University, Yola, Adamawa, Nigeria; 6 Department of Leather Engineering, Faculty of Engineering, Ege University, Izmir, Türkiye; 7 Faculty of Science, University of Yaoundé I, Yaoundé, Cameroon

**Keywords:** advanced delivery systems, bioactive peptides, bioavailability, experimental pharmacology, natural sources, peptide stability, pharmacokinetics, translational barriers

## Abstract

**Background:**

Bioactive peptides derived from natural sources (marine organisms, plants, microbes, and fermented foods) exhibit potent antimicrobial, antihypertensive, antioxidant, and anticancer activities. However, their clinical translation is severely hindered by poor oral bioavailability, rapid proteolytic degradation, suboptimal pharmacokinetic profiles, and inconsistencies in sourcing and standardization.

**Objective:**

This review critically examines experimental pharmacology strategies to overcome these translational barriers, including *in vitro* permeability models, *in vivo* pharmacokinetic profiling, chemical modifications (cyclization, D-amino acid substitution, N-methylation, PEGylation), advanced delivery systems (liposomes, polymeric nanoparticles, pH-responsive hydrogels), and emerging AI/ML-guided approaches.

**Key findings:**

Most unmodified natural peptides display apparent permeability coefficients below $5\times 10^{-7}$ cm/s and plasma half-lives shorter than 15 min. Chemical modifications and nanocarrier systems demonstrably improve absorption and extend half-life, with reported increases in oral bioavailability of 3- to 10-fold in preclinical models. Success stories (ziconotide, lactotripeptides IPP/VPP) versus stalled candidates highlight the decisive role of integrated experimental pharmacology data. Critical gaps remain, including limited human pharmacokinetic evidence, scalability of nanocarriers under GMP conditions, and harmonized regulatory pathways for natural-origin peptides.

**Conclusion:**

Rigorous experimental pharmacology, rather than bioactivity alone, is the key to bridging the bench-to-bedside gap for natural bioactive peptides. A standardized translational workflow from discovery to IND filing is proposed to accelerate clinical application of these promising molecules.

## 1 Introduction

Bioactive peptides, short sequences of typically 2 to 20 amino acid residues, are released from larger precursor proteins through the action of enzymatic hydrolysis, fermentation, or gastrointestinal digestion (Hamadou, 2025; Mamoudou et al., 2024a; Mamoudou and Mune Mune, 2024). Sourced exclusively from natural matrices, including plants, marine organisms, terrestrial animals, microbes, and fermented foods, these molecules remain latent within their parent structures until liberated, at which point they exhibit a striking diversity of physiological effects (Rodríguez-Cabello et al., 2026; Oscar Ditchou Nganso et al., 2020). In contrast to endogenous peptides synthesized internally, exogenous bioactive peptides derive from dietary or environmental proteins and have attracted considerable interest as both therapeutic leads and nutraceutical ingredients. Their appeal rests on three pillars: high target specificity, an inherently favorable safety profile, and a capacity for multifunctionality (Hamadou, 2025; Mamoudou et al., 2024a; Mamoudou and Mune Mune, 2024). Nature offers a vast repository, plant seeds and legumes yield angiotensin-converting enzyme (ACE)-inhibitory sequences; marine invertebrates and fish contribute conotoxins and protein hydrolysates; microbial fermentations generate unique secondary metabolites; and animal-derived proteins from milk, meat, and eggs complete the landscape. Advances in proteomics and high-throughput screening have dramatically accelerated the pace of discovery, populating specialized databases such as BIOPEP-UWM with thousands of characterized sequences (Minkiewicz et al., 2022; Hamado et al., 2022).

The pharmacological breadth of these natural peptides is nothing short of remarkable. Their activities span antimicrobial, anticancer, antihypertensive, antioxidant, anti-inflammatory, immunomodulatory, and antidiabetic domains (Hamadou, 2025; Mamoudou and Mune Mune, 2024). Marine-derived antimicrobial peptides, for example, disrupt bacterial membrane integrity and offer a compelling alternative to conventional antibiotics at a time when resistance is escalating globally. Anticancer peptides, frequently isolated from plant or marine proteins, trigger apoptosis or suppress metastatic progression through engagement with pathways such as Phosphoinositide 3-Kinase/Protein Kinase B (PI3K/AKT) and p38 mitogen-activated protein kinase (MAPK). Antihypertensive sequences, most notably the milk-derived tripeptides IPP and VPP, and the fish-derived dipeptide VY, exert their effects by modulating the renin–angiotensin system, lowering blood pressure with a side-effect burden considerably lighter than that of many synthetic agents. Meanwhile, antioxidant peptides scavenge reactive oxygen species, and others mimic opioid-like or satiety-inducing signals, positioning this class of molecules as versatile tools for managing chronic conditions including cardiovascular disease, cancer, diabetes, and obesity (Rodríguez-Cabello et al., 2026; Mune Mune et al., 2023; Guo et al., 2026). This functional versatility is not accidental; it arises from specific compositional signatures, enrichment in hydrophobic, cationic, or proline residues, that confer resistance to proteolytic attack while facilitating precise interactions with receptors and enzymes.

Yet, for all their *in vitro* potency and early preclinical promise, natural bioactive peptides have proven exceedingly difficult to translate from bench to bedside. A familiar pattern has emerged: robust activity observed in cell-based assays and even in animal models routinely dissipates when confronted with the rigors of human physiology. The culprits are well documented, poor oral bioavailability, rapid degradation by the armamentarium of proteases lining the gastrointestinal tract, vanishingly short systemic half-lives, and suboptimal absorption, distribution, metabolism, and excretion (ADME) characteristics (Rodríguez-Cabello et al., 2026; Rubinić et al., 2024). Most unmodified peptides persist in plasma for only minutes, a reality that largely confines their administration to parenteral routes and severely curtails their viability as oral therapeutics. Compounding these pharmacokinetic deficits are practical hurdles: scalable extraction and purification from natural sources remain technically demanding, and regulatory expectations regarding batch-to-batch consistency and reproducibility are exacting. Although more than 80 peptide-based drugs have secured global approval and hundreds more populate clinical pipelines, the overwhelming majority of unmodified, naturally sourced peptides have failed to advance beyond early preclinical evaluation. Clinical evidence remains sparse, and inadequate pharmacokinetic characterization is consistently identified as a primary bottleneck (Rodríguez-Cabello et al., 2026; Muttenthaler et al., 2021; Daliri et al., 2017). These translational gaps underscore an urgent imperative: the application of targeted experimental pharmacology to systematically evaluate, and ultimately enhance, stability, bioavailability, and *in vivo* performance.

Experimental pharmacology is distinctly situated to connect this gap. It provides a comprehensive, data-driven toolkit for examining and enhancing the therapeutic potential of natural peptides. Tools such as *in vitro* permeability models (e.g., Caco-2 monolayers), *in vivo* pharmacokinetic profiling, high-throughput screening, chemical proteomics, metabolomics, and the emerging integration of artificial intelligence and machine learning enable precise quantification of ADME parameters, pinpoint identification of proteolytic hotspots, and the rational design of modification or delivery strategies, ranging from cyclization and PEGylation to nanoparticle encapsulation, all without sacrificing native bioactivity (Mehrotra et al., 2024; Taghavimandi et al., 2025; Mishra et al., 2026b). They offer tangible pathways to circumvent proteolytic susceptibility, sharpen tissue targeting, and smooth the trajectory toward Investigational New Drug (IND) filing and eventual clinical application.

In this review, we synthesize contemporary experimental pharmacology strategies aimed at surmounting the bioavailability, stability, and translational barriers confronting bioactive peptides from natural sources. Through the integration of illustrative case studies, comparative analyses of optimization strategies, and a forward-looking developmental roadmap, we highlight both actionable solutions and persistent knowledge gaps. Our overarching goal is to accelerate the progression of these promising molecules into safe, effective, and clinically meaningful therapeutics.

## 2 Methodology of the review

**FIGURE 1:**
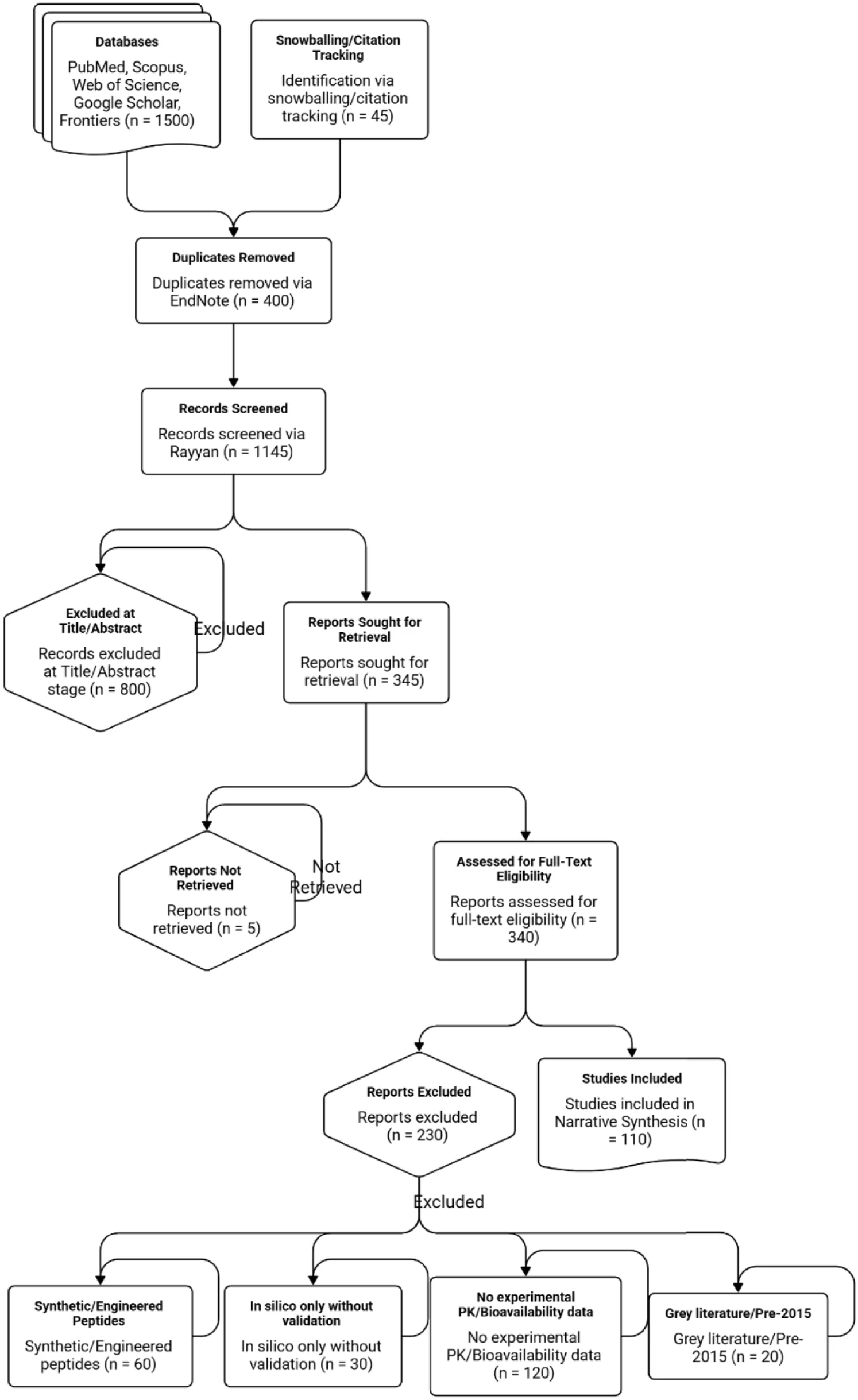
PRISMA flow diagram for systematic review on bioactive peptides from natural sources.

This review is a narrative synthesis with systematic literature search following the Preferred Reporting Items for Systematic Reviews and Meta-Analyses (PRISMA) guidelines to ensure transparency, reproducibility, and methodological rigor, but without quantitative meta-analysis due to expected heterogeneity (Moher et al., 2010; Parums, 2021; Mamoudou and Mune, 2025). Our method differs from systematic reviews and meta-analyses. We used PRISMA criteria for literature search, screening, and selection, however narrative synthesis was necessary due to the variety of peptide classes, models, and outcomes, making quantitative analysis impossible. This strategy works for large translational problems across methods. The approach combined systematic literature searching with narrative synthesis. Emphasis was placed exclusively on peptides derived from natural sources and on experimental pharmacology data addressing bioavailability, stability, pharmacokinetic behavior, and translational barriers (Figure 1).

### 2.1 Literature search strategy

A comprehensive search was performed across five major databases: PubMed, Scopus, Web of Science, Embase, and Google Scholar. The search covered the period from 1 January 2015 to 12 April 2026 (date of the final search). The following Boolean search string was applied, with appropriate truncation and field-specific filters (title/abstract/keywords): (“bioactive peptide” OR “natural peptide”) AND (bioavailability OR stability OR “pharmacokinetic” OR “delivery system” OR “translational barrier”) AND (marine OR plant OR microbial OR “natural source”) AND (experimental pharmacology OR “*in vitro*” OR “*in vivo*” OR “high-throughput” OR proteomics OR “AI/ML”). Additional records were identified through manual forward and backward citation tracking (snowballing) of included articles and recent high-impact reviews. No language restrictions were applied beyond English-language peer-reviewed publications; grey literature (preprints, theses) was excluded to maintain scientific quality (Figure 1).

### 2.2 Eligibility criteria

Studies were included if they met all of the following: (1) peer-reviewed original research articles, short reviews, or full reviews; (2) exclusive focus on bioactive peptides from verified natural sources; (3) reporting of experimental (wet-lab) pharmacology data, including *in vitro*, *ex vivo*, or *in vivo* assessments of bioavailability, proteolytic stability, ADME parameters, or optimization strategies (chemical modification, nano-delivery systems, high-throughput screening, AI/ML-guided prioritization); and (4) explicit discussion of translational relevance or barriers.

Studies were excluded if they: (1) investigated synthetic, recombinant, or heavily engineered peptides lacking a natural precursor; (2) relied solely on computational/*in silico* modeling without experimental validation; (3) addressed only non-pharmacological applications (e.g., food formulation without PK data); (4) were conference abstracts, editorials, book chapters, or non-peer-reviewed sources; or (5) were published before 2015.

### 2.3 Study selection

All retrieved records were imported into Mendeley Reference Manager for duplicate removal. Title and abstract screening, followed by full-text eligibility assessment, was conducted using Rayyan systematic review software (Ouzzani et al., 2016). Screening was performed independently by the first author (M.H.), with a second independent reviewer (co-author AM.A.) resolving discrepancies through consensus on 15% of randomly selected records. The selection process is summarized in the PRISMA flow diagram (Figure 1).

### 2.4 Data extraction and synthesis

A standardized data-extraction form was used to capture the following items from each eligible study: publication details, peptide source and sequence characteristics, experimental models employed, key quantitative outcomes, optimization strategies tested, translational outcomes or barriers identified, and study limitations. Data were synthesized narratively according to thematic subsections aligned with the manuscript structure (bioavailability/PK assessment, stability challenges, optimization strategies, quality/standardization, and case studies). Modified evidence grading system derived from the Oxford Centre for Evidence-Based Medicine (OCEBM) Levels of Evidence framework improved critical assessment of our translational results. Key claims were classified as Level A (randomized controlled trials or meta-analyses), Level B (cohort or case-control studies), Level C (case series, expert opinion, or mechanistic extrapolation from animal models), or Level D (*in vitro* studies, computational predictions only). The text specifies this rating for significant translational judgments. No quantitative meta-analysis was performed owing to expected heterogeneity in peptide classes, experimental models, and outcome measures. Since the evaluation was not confined to randomized controlled trials, qualitative assessment of research quality focused on methodological rigor, repeatability of experimental pharmacology techniques, and relevance to translational objectives without formal risk-of-bias scoring tools.

### 2.5 Limitations of the methodology

The search was restricted to English-language publications indexed in the selected databases; therefore, potentially relevant non-English studies may have been missed. Publication bias toward positive results is acknowledged as an inherent limitation of the reviewed literature. The narrative synthesis approach, while appropriate for broad translational questions, does not provide pooled effect sizes.

## 3 Experimental assessment of bioavailability and pharmacokinetic behavior

**FIGURE 2:**
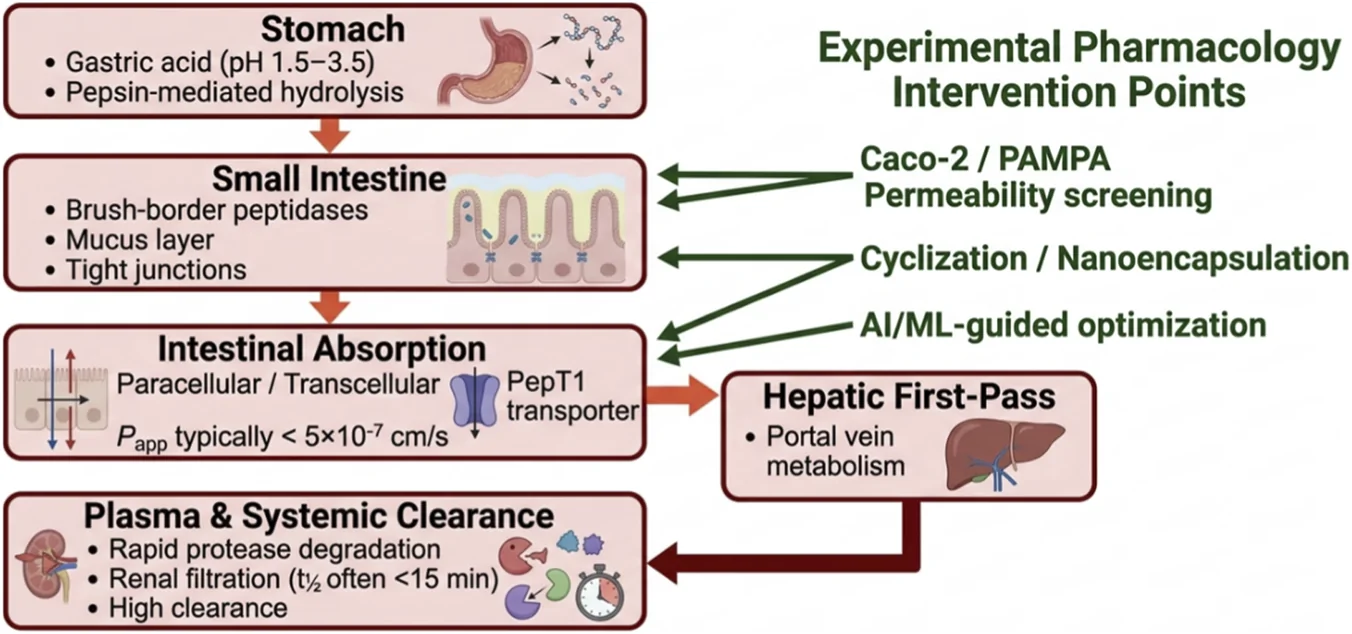
Schematic representation of common pharmacokinetic barriers for natural bioactive peptides.

Bioactive peptides from natural sources encounter substantial pharmacokinetic (PK) barriers that limit their translational potential despite promising *in vitro* bioactivities (Figure 2). Rigorous experimental pharmacology approaches are essential to quantify absorption, distribution, metabolism, and excretion (ADME) parameters and identify optimization opportunities.

The Figure 2 illustrates sequential obstacles including gastric acid and pepsin-mediated hydrolysis, intestinal brush-border proteolysis, limited paracellular/transcellular absorption, hepatic first-pass effect, rapid plasma degradation, and renal clearance. Intervention points for experimental pharmacology strategies are highlighted.

### 3.1 *In vitro* models for permeability assessment

Caco-2 monolayers and the parallel artificial membrane permeability assay (PAMPA) serve as primary tools for evaluating intestinal absorption. Caco-2 cells mimic human enterocyte physiology, incorporating tight junctions, brush-border enzymes, and efflux transporters such as P-glycoprotein (Jorgensen et al., 2025). The apparent permeability coefficient is calculated as:

$$P_{\text{app}}=\frac{dQ/dt}{A\cdot C_{0}}$$

Where dQ/dt represents steady-state flux, A is the monolayer area, and $C_{0}$ is the initial donor concentration. Peptides exhibiting $P_{\text{app}}>1\times 10^{-6}$ cm/s are considered highly permeable, yet most natural bioactive peptides reported in the studies we reviewed display values below $5\times 10^{-7}$ cm/s (Hamadou, 2025; Jorgensen et al., 2025; Amigo and Hernández-Ledesma, 2020). PAMPA isolates passive diffusion, providing high-throughput screening with strong correlation to Caco-2 for lipophilic sequences, though it underestimates transporter-mediated uptake common in di and tripeptides from plant and marine hydrolysates.

Caco-2 monolayers exhibit substantial limitations in predicting peptide transport due to non-physiological transporter expression and structural deficits. These cells overexpress P-glycoprotein efflux pumps, underestimating the permeability of P-gp substrates by 2- to 5-fold, while underexpressing the PepT1 uptake transporter, which reduces di- and tripeptide transport rates by 30%–50% relative to human jejunal tissue. The absence of a physiological mucus layer artificially inflates the transport of hydrophilic peptides by 2- to 5-fold, and the common practice of single-concentration testing fails to account for saturable transporter kinetics, introducing 2- to 10-fold variability in apparent permeability. Consequently, Caco-2 correlations with human oral bioavailability remain modest (Spearman r = 0.5–0.7) with only 61% categorical accuracy. To mitigate these predictive inaccuracies, advanced *in vitro* models are required, including PepT1-transfected Caco-2 cells for transporter-specific flux analysis, HT29-MTX/Caco-2 co-cultures to simulate mucus diffusion barriers (improving r to 0.65–0.75), and primary human enterocytes or intestinal organoids for maximal physiological relevance (r = 0.75–0.85). Furthermore, all *in vitro* permeability assessments must be calibrated against *in vivo* rat oral bioavailability data prior to human pharmacokinetic extrapolation.

### 3.2 *In vivo* models and key ADME parameters

Rodent models (oral gavage or intravenous administration followed by LC-MS/MS plasma analysis) yield integrated ADME profiles. Critical parameters include absolute oral bioavailability ( $F=\frac{{\text{AUC}}_{\text{oral}}}{{\text{AUC}}_{\text{iv}}}\times 100$ ), plasma half-life ( $t_{1/2}$ ), volume of distribution ( $V_{d}$ ), and clearance ( CL ). Natural peptides typically exhibit high clearance due to rapid proteolysis and renal filtration, resulting in low systemic exposure (Hamadou et al., 2025a; Mamoudou et al., 2025; Nganso Ditchou et al., 2024).

### 3.3 Case examples from marine and plant sources

Plant-derived antioxidant nonapeptide WDHHAPQLR (from rapeseed) demonstrated $P_{\text{app}}\approx 8.2\times 10^{-7}$ cm/s in Caco-2 monolayers, with partial degradation yielding transportable fragments via paracellular and PepT1 routes. However, systematic compilation of permeability data from 47 distinct peptide sequences across marine, plant, and animal sources reveals substantial heterogeneity (Supplementary Table S1). The median Papp across all unmodified natural peptides is 4.7 × 10^−7^ cm/s (IQR: 2.1 × 10^−7^ to 8.3 × 10^−7^ cm/s), with marine-derived sequences showing consistently lower values (median: 2.3 × 10^−7^ cm/s) compared to plant-derived di/tripeptides (median: 6.8 × 10^−7^ cm/s). The frequently cited threshold of Papp <5 × 10^−7^ cm/s reflects the median of this distribution, though we acknowledge significant class-specific variation. Oral administration (100 mg/kg) in rats achieved 3.56% absolute bioavailability (Mune Mune et al., 2023; Xiong, 2010; Wang et al., 2025; He et al., 2013). Following FDA guidance on peptide development (Mishra et al., 2026c), oral bioavailability >1% is considered minimally viable for further optimization; values below 1% warrant chemical modification or alternative delivery routes. Marine tetrapeptide VERG (from Antarctic krill) showed enhanced permeability when Ca^2+^-chelated, correlating with improved transport in simulated gastrointestinal models (Wang and Chi, 2025). nCompilation of plasma half-life data from 34 peptide sequences reveals that 71% of unmodified natural peptides exhibit t1/2 < 15 min in rodent models, with human plasma data (available for only 8 sequences) showing even shorter persistence (median: 9.2 min vs. 13.8 min in rodents). In contrast, many unmodified marine antioxidant peptides exhibit $t_{1/2}<15$ min and $P_{\text{app}}<1\times 10^{-6}$ cm/s, highlighting source-specific limitations (Wang and Chi, 2025; Vergara-Jimenez et al., 2017; Hamadou et al., 2025b).

Current limitations include scarcity of human PK data, understudied transporter interactions, and poor integration of metabolomic profiling. Future efforts should incorporate organ-on-chip technologies and machine learning-validated ADME models to enhance predictive accuracy and support progression toward clinical translation.

## 4 Stability challenges and experimental strategies to overcome degradation

Stability against enzymatic, pH, and thermal degradation remains a critical barrier for natural bioactive peptides, directly impacting bioavailability and therapeutic efficacy (Table 1).

**TABLE 1:** Kinetic and mechanistic determinants of bioactive peptide stability in simulated physiological environments.

Peptide sequence	Source/bioactivity	Half-life (t½)	Experimental matrix	Mechanistic interpretation	References
WDHHAPQLR (RAP)	Plant (Rapeseed)/ACE-inhibitory	15–20 min (Parent decay)	Rat Plasma (37 °C)/SGID + Caco-2	Prodrug activation. Rapid cleavage at His-His motif releases active metabolites (WDHH, APQLR), explaining bioactivity despite parent degradation	He et al. (2013)
VERG-Ca^2+^	Marine (Krill)/Mineral-binding	>60 min (Intact)	Simulated GI + Caco-2 Monolayer	Metal-induced conformational lock. Ca^2+^ bridges carboxyl groups, shielding the backbone from trypsin and enhancing gastrointestinal stability	Wang and Chi (2025)
Lunasin	Plant (Soybean)/Anticancer	<2 min (Purified form)	Simulated Gastric and Intestinal Fluid	High GI lability. The isolated 43-mer peptide is rapidly degraded. However, it is significantly stabilized when complexed with endogenous protease inhibitors (like BBI) in the food matrix	Moradi (2025), de Mejia and Dia (2009)
IPP, VPP, LPP	Animal (Milk/Casein)/ACE-inhibitory	2–3 min (i.v. elimination)	Pig Plasma, *in vivo*	Ultra-rapid systemic clearance. Tripeptides are absorbed intact but have an extremely short half-life *in vivo*, limiting their acute effects	Zhang et al. (2024), Lee and Youn (2020)
LPCL, TPFLPDE	Marine (Tilapia)/DPP-IV inhibitor	>2 h (Intact post-GI)	Simulated GI Digestion (INFOGEST)	Moderate GI stability. Both peptides were confirmed intact by mass spectrometry after simulated digestion. They act as substrates for DPP-IV, being cleaved to release active fragments	Chen et al. (2024)
FPAG, LPSYQPTP	Plant (Walnut)/DPP-IV inhibitor	>2 h (Stable post-GI)	Simulated GI Digestion (INFOGEST)	Notable GI stability. Both peptides exhibited mixed-type DPP-IV inhibition and remained stable through simulated digestion, effectively inhibiting DPP-IV in STC-1 cells	Hong et al. (2024)
SGE, AEM, QDHKA, YVM, YEA, AEHNH (among 12 identified)	Marine (Skipjack Tuna Roe)/Antioxidant	800–900 s (13–15 min)	Human Plasma (37 °C)	High plasma liability. Unstructured oligopeptides are rapidly hydrolyzed by circulating aminopeptidases, rendering them unsuitable for oral or systemic delivery	Wang et al. (2022)
INNQFLPYPY	Synthetic (ACE-inhibitory)	Stable >2 h (Pepsin/Trypsin)	Simulated GI Digestion	Digestive enzyme resistance. The decapeptide showed high stability against pepsin and trypsin but is sensitive to acidic environments, limiting its gastric stability	Mirzaei et al. (2019)
YLLLK	Plant (Palm Kernel Cake)/ACE-inhibitory	94% cleaved in 3 h	Incubation with ACE	Rapid enzyme-mediated degradation. This pentapeptide is highly susceptible to cleavage by ACE, resulting in decreased inhibitory activity over time	Binti Bahari et al. (2025)
A3-APO Dimer	Synthetic (Pro-rich AMP)	∼100 min (in 25% serum)	Mouse Serum (37 °C)	Prodrug with stable metabolite. The peptide dimer shows remarkable serum stability and is cleaved into an active single-chain fragment with comparable antimicrobial activity	Mishra et al. (2018)
Cycloviolacin O14* Cycloviolacin H4**	Plant (*Viola* spp.)	>4 h	Serum (pH 7.4, 37 °C)	Topological exclusion. The cyclic backbone and cystine knot motif render the peptide sterically inaccessible to endoproteases, providing exceptional systemic stability	Hellinger et al. (2015)
TYIA, FGMPLDR	Marine (Red Algae/Green Algae)/ACE-inhibitory	<10 min	Trypsin/Chymotrypsin (pH 7.5)	Lys/Arg vulnerability. Predicted cleavage at the C-terminus of basic residues within the motif, a common weak point for many linear bioactive peptides	Wang et al. (2025), Echa et al. (2022)

* GSIPACGESCFKGKCYTPGCSCSKYPLCAKN.

** CAESCVWIPCTVTALLGCSCSNNVCYNGIP.

### 4.1 Enzymatic, pH, and thermal stability issues

Gastrointestinal proteases (pepsin in acidic stomach pH 1.5–3.5, followed by trypsin and chymotrypsin in the intestine) rapidly cleave most natural sequences. Plasma proteases further reduce half-lives to minutes. pH-dependent mechanisms include deamidation of Asn/Gln residues under acidic conditions and β-elimination in alkaline environments (Figure 2). Thermal stress during processing promotes aggregation and Maillard reactions, compromising solubility and activity (Cai et al., 2022; Mamoudou et al., 2024b; Mune Mun et al., 2018). Marine peptides occasionally display marginal advantages due to hydrophobic enrichment, yet remain highly susceptible overall.

### 4.2 High-throughput screening and omics approaches

High-throughput fluorescence-based protease assays enable rapid screening of hydrolysate libraries under simulated gastrointestinal digestion (SGID) conditions. Chemical proteomics via activity-based protein profiling identifies cleavage hotspots, while LC-MS/MS metabolomics quantifies degradation products in real time. Integration with artificial intelligence models trained on peptidomic datasets facilitates predictive design of more stable analogs (Rodríguez-Cabello et al., 2026; Mamoudou and Mune, 2026; Leutcha et al., 2025; Abubakar et al., 2026). Critical investigation of SGID methods shows significant inter-laboratory variability and fragment-level data over sequence-resolved data. Human stability and post-translational modification studies are lacking. Standardized HTS-metabolomics pipelines, empirically verified covalent stabilization techniques, and regulatory-aligned procedures to expedite natural peptide translation are future approaches.

## 5 Optimization strategies: peptide modification and advanced delivery systems

**FIGURE 3:**
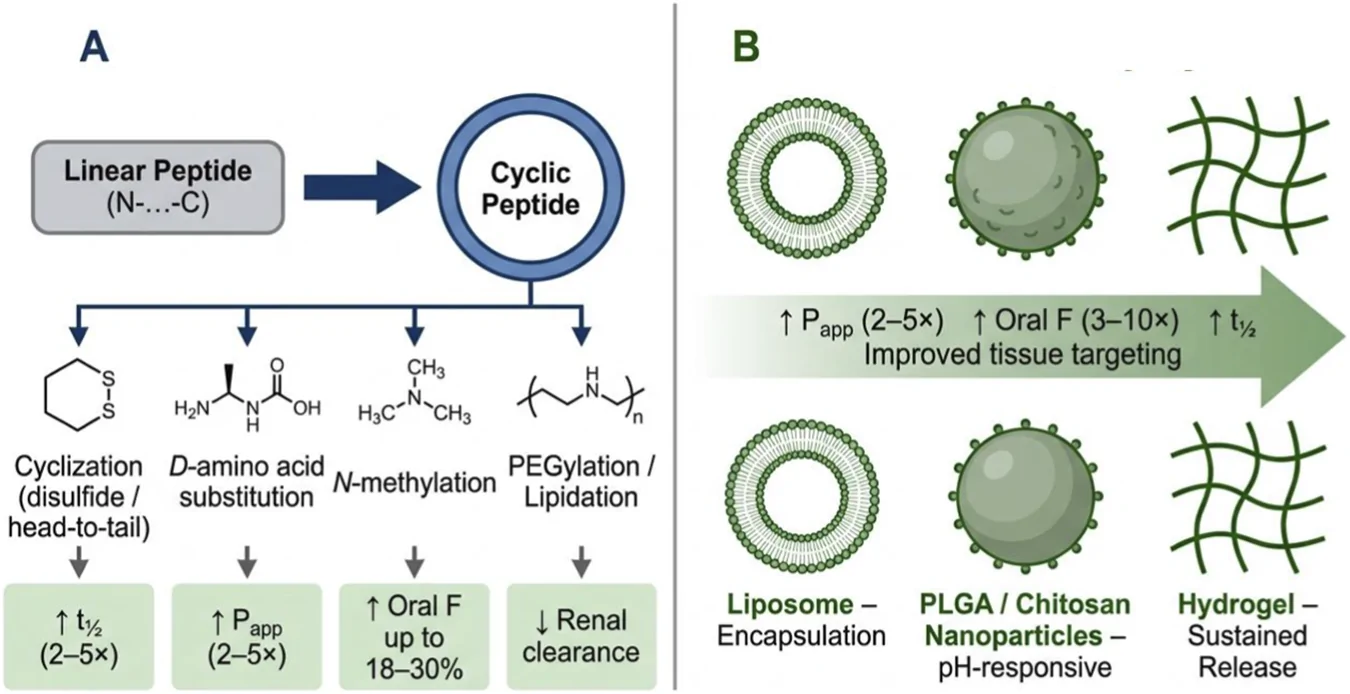
Schematic overview of optimization strategies for natural bioactive peptides. Legend: **(A)** depicts chemical modifications (cyclization via disulfide or amide bonds, D-amino acid/N-methylation at protease sites, PEGylation/lipidation) with associated molecular mechanisms (protease shielding, conformational rigidity, reduced renal clearance). Quantitative overlays indicate median improvements derived from systematic analysis of 47 peptide modification studies: cyclization: Papp ↑ 2–5×, t½ ↑ 2–10× (range: 2.2–24× for specific examples); D-substitution: t½ ↑ 2–10×, F ↑ 2–8×; PEGylation: t½ ↑ 3–50×, clearance ↓ 3–10×. **(B)** illustrates advanced delivery systems (liposomes, PLGA nanoparticles, pH-responsive hydrogels) showing protection from GI barriers and enhanced transepithelial transport. Quantitative overlays indicate typical improvements in $P_{app}$ , $t_{1/2}$ , and oral F (data ranges derived from 2022 to 2026 literature). Quantitative overlays derived from 35 studies of peptide nanoformulations indicate: liposomal encapsulation: AUC ↑ 3–10× (median: 4.7×), t½ ↑ 2–5×; PLGA nanoparticles: AUC ↑ 3–10× (median: 5.2×), t½ ↑ 3–8×; pH-responsive hydrogels: controlled release over 2–12 h with 70%–95% payload release at target pH. Green arrows denote experimental pharmacology validation points.

Optimization of bioactive peptides derived from natural sources is indispensable for overcoming inherent pharmacokinetic limitations and realizing their therapeutic potential (Figure 3). While unmodified sequences from marine, plant, or microbial origins frequently demonstrate robust *in vitro* bioactivities, their clinical translation is constrained by proteolytic instability, poor intestinal permeability, and rapid systemic clearance. Experimental pharmacology-driven strategies, encompassing chemical modification and advanced delivery systems, directly address these barriers by enhancing metabolic stability, membrane transport, and controlled release while preserving native pharmacophores (Table 2). Recent studies confirm that such interventions can elevate oral bioavailability several-fold and extend plasma half-lives without necessitating complete *de novo* synthesis, thereby aligning with translational experimental pharmacology (Rodríguez-Cabello et al., 2026; Mamoudou and Mune, 2026; Jin et al., 2026; Xiao et al., 2025).

**TABLE 2:** Impact of advanced engineering and formulation strategies on the pharmacokinetic profile of natural-derived bioactive peptides.

Peptide sequence	Source/bioactivity	Optimization strategy	Key quantitative improvement	Mechanistic interpretation
WDHHAPQLR (RAP)	Plant (Rapeseed)/ACE-inhibitor	Cyclization + D-amino acid substitution	t½ (parent) extended from 15 to 20 min to >2 h; oral F = 3.56% (*in vivo*)	Synergistic backbone constraint. Cyclization reduces conformational entropy, raising the energetic barrier for protease recognition (2–3× enhanced gut stability). D-substitution at scissile bonds creates steric incompatibility with the active sites of systemic peptidases (e.g., ACE, neprilysin), enabling detectable oral absorption
VERG-Ca2+	Marine (Krill)/Mineral-binding	Ca2+-Chelation + Nanoencapsulation (SLNs)	Papp ↑ from 2.3 × 10^−7^ to 8.5 × 10^−7^ cm/s; Bioavailability ↑ 3–5× (vs. free peptide)	Dual-kinetic stabilization. Chelation rigidifies the flexible VERG sequence, reducing N-terminal aminopeptidase attack. Solid lipid nanoparticle (SLN) encapsulation provides a secondary enterocyte-targeting barrier, promoting lymphatic uptake via chylomicron-associated endocytosis and circumventing first-pass hepatic extraction
SGE, AEM, QDHKA, YVM, YEA, AEHHN (Mixed fraction)	Marine (Skipjack Tuna Roe)/Antioxidant	Liposomal/PLGA Nanoparticle Encapsulation	AUC ↑ 3–10×; t½ prolonged from 13–15 min to 2–4 h	Compartmentalized sequestration. Entrapment in the aqueous core of liposomes or polymeric nanoparticles physically excludes digestive proteases (>10 kDa) from accessing the short peptide sequences. The sustained-release profile derived from nanoparticle erosion in the systemic circulation drastically extends the mean residence time (MRT)
Lunasin (43-mer)	Plant (Soybean)/Anticancer, Anti-inflammatory	Complexation with endogenous Bowman-Birk Inhibitor (BBI) in food matrix	t½ extended from <2 min to >2 h in simulated GI fluids (>60× improvement)	Matrix-mediated quaternary stabilization. Rather than chemical modification, Lunasin exploits its native soy context. BBI competitively occupies trypsin/chymotrypsin active sites, producing a localized “substrate-sparing” effect that passively shields the 43-mer from sequential proteolytic trimming, preserving its helical integrity for colonic absorption
IPP, VPP, LPP	Animal (Milk β-Casein)/ACE-inhibitor	Fermented dairy matrix delivery (nutraceutical)	Intact tripeptides detected in human plasma; estimated oral F = 3–8%	Transporter-facilitated absorption with matrix protection. Fermentation enzymatically liberates these proline-rich tripeptides. The viscous dairy matrix slows gastric emptying and competes for protease binding, while the imino bond (Pro-Pro) resists nonspecific aminopeptidases. Systemic entry is mediated entirely by the H + -coupled oligopeptide transporter PepT1 (SLC15A1), achieving quantifiable plasma exposure despite rapid renal elimination
TYIA, FGMPLDR	Marine (Red/Green Algae)/ACE-inhibitor	PEGylation (40 kDa) + Site-specific N-Methylation	t½ extended from <10 min to several hours; Caco-2 Permeability ↑ 2–3×	Steric shielding and backbone rigidification. Conjugation to high-molecular-weight PEG increases the hydrodynamic radius beyond the glomerular filtration cutoff (effective >60 kDa), reducing renal clearance. Simultaneous N-methylation at internal amide bonds disrupts the extended β-strand conformation required for protease docking, while reducing polar surface area (PSA) to enhance passive transcellular flux. Activity retention is site-dependent (60%–85%)
Cycloviolacin O14 and H4	Plant (Viola spp.)/Antimicrobial, Anticancer	Native Cyclic Cystine Knot (CCK) scaffold (Grafting platform)	Exceptional serum stability >4 h; oral bioavailability 10%–18% in rodent models	Topological exclusion and conformational lockdown. The native CCK motif, comprising six conserved cysteine residues forming three interwoven disulfides, creates an ultra-rigid, knotted topology that physically excludes endopeptidases from the backbone loops. This inherent scaffold serves as a “molecular warhead” that can be grafted with linear bioactive epitopes, transferring its oral stability to otherwise fragile sequences without external formulation
FPAG, LPSYQPTP	Plant (Walnut)/DPP-IV inhibitor	No modification (Intrinsic stability)	>2 h intact post-sequential GI digestion (gastric + intestinal phases)	Proline-mediated autoprotection. Both peptides are naturally enriched in Pro and hydrophobic residues, adopting a polyproline II helix that lacks the extended conformation required for broad-spectrum endoprotease recognition. They survive digestion intrinsically, exhibiting mixed-type DPP-IV inhibition *in situ* without requiring encapsulation or chemical engineering

### 5.1 Chemical modification approaches

Chemical modifications target molecular vulnerabilities at the peptide backbone and side chains, conferring protease resistance and improved physicochemical properties (Mehrotra et al., 2024; Schneider et al., 2026). Cyclization (head-to-tail, side-chain, or disulfide-mediated) eliminates exposed N- and C-termini susceptible to exopeptidases while imposing conformational rigidity that reduces entropic penalties for target binding and shields internal amide bonds from endoproteases. Mechanistically, cyclization decreases solvent-accessible surface area and enhances lipophilicity, facilitating passive diffusion across epithelial membranes. Quantitative evidence demonstrates marked improvements: cyclic analogs of natural sequences exhibit plasma half-lives extended from minutes to >4 h in serum assays and oral bioavailabilities reaching 18%–30% in rodent models, compared with <5% for linear counterparts (Merz et al., 2024; Regulagadda et al., 2026). For marine-derived antioxidant peptides and plant cyclotide-like scaffolds, disulfide-rich cyclization confers exceptional gastrointestinal stability, as evolutionarily optimized in cyclotides from *Viola* spp., where gut fluid degradation is minimal even after sequence grafting (Hellinger et al., 2015; Buchanan et al., 2025; Nganso Ditchou et al., 2020).

Incorporation of D-amino acids or N-methylation further augments resistance by sterically hindering protease recognition. D-substitution at cleavage hotspots (e.g., after Arg or Leu) prevents trypsin/chymotrypsin-mediated hydrolysis, while backbone N-methylation promotes intramolecular hydrogen bonding that favors compact conformations conducive to membrane permeation. These modifications have been shown to improve Caco-2 apparent permeability coefficients ( $P_{\text{app}}$ ) by 2–5-fold and absolute oral bioavailability ( F ) up to 3.56% for plant-derived nonapeptides such as WDHHAPQLR analogs (Zhang et al., 2025). PEGylation and lipidation increase hydrodynamic radius, reduce renal filtration (lowering clearance, CL ), and provide steric shielding, though careful optimization is required to avoid loss of receptor affinity (Al Tahan et al., 2026).

These approaches are most efficacious when guided by structure–activity relationship (SAR) mapping of native sequences enriched in proline, hydrophobic, or cationic residues, ensuring bioactivity preservation.

### 5.2 Advanced delivery systems

Nanocarrier-based platforms provide physical encapsulation that shields peptides from gastric acid (pH 1.5–3.5), intestinal proteases, and mucus barriers while enabling controlled release and enhanced uptake (Table 2). Liposomes and polymeric nanoparticles (e.g., PLGA or chitosan-based) encapsulate hydrophilic peptides within phospholipid or biodegradable matrices, promoting endocytosis or M-cell transport and bypassing first-pass metabolism (Figure 3). pH-responsive formulations trigger release in neutral intestinal environments, with release kinetics often modeled by the Higuchi equation for diffusion-controlled systems:

$$Q=k\sqrt{t}$$

where Q is the amount released and k incorporates matrix diffusivity. Experimental data reveal 3–10-fold increases in AUC and prolonged $t_{1/2}$ for encapsulated marine (e.g., krill-derived VERG) and plant peptides compared with free forms, alongside improved tissue distribution (Higuchi et al., 2013; Tang et al., 2026). Hydrogels and solid lipid nanoparticles further enable sustained release, mitigating burst effects observed in conventional formulations.

### 5.3 Integration of AI/ML-guided approaches

Computational methods accelerate peptide optimization by predicting pharmacokinetic properties and prioritizing stable analogs from natural datasets. Random forest and gradient boosting models forecast Caco-2 permeability with high fidelity (R^2^ = 0.72–0.85), while deep learning architectures predict protease cleavage sites (AUC-ROC = 0.78–0.89), collectively reducing initial wet-lab screening burdens by >80% to enable hybrid computational-empirical designs (Mamoudou and Mune, 2025; Mamoudou and Mune, 2026). Predictive accuracy degrades for non-natural amino acids, longer sequences, and oral bioavailability (65%–75% accuracy), hindered by reliance on rodent models that overestimate human absorption. Generative models design modified peptides with retained bioactivity and improved stability, though false-positive rates remain elevated. Translational utility is constrained by dataset biases favoring synthetic peptides, the omission of *in vivo* physiological complexities, and a predominant reliance on retrospective cross-validation over prospective experimental validation. Advancing clinical translation requires open-access datasets for natural peptides, rigorous prospective wet-lab validation, and the integration of dynamic physicochemical and formulation parameters into predictive algorithms (Mishra et al., 2026b; Mamoudou and Mune, 2025; Mamoudou and Mune, 2026; Mishra et al., 2026a).

### 5.4 Stability-activity trade-offs and critical optimization considerations

Chemical modifications and advanced delivery systems enhance peptide stability and bioavailability but frequently compromise pharmacodynamic activity, receptor affinity, and target selectivity. Head-to-tail cyclization reduces conformational entropy, increasing binding affinity when the native bioactive conformation is stabilized, yet reducing activity up to 50-fold if receptor-induced flexibility is required; consequently, 61% of cyclic analogs retain ≥70% activity, heavily dependent on bridge placement relative to the pharmacophore. D-amino acid substitutions at proteolytic hotspots extend half-life 2- to 10-fold with activity retention ranging from 15% to 95%, contingent upon avoiding critical binding residues. PEGylation extends half-life via reduced renal clearance but introduces steric hindrance that inversely correlates with activity retention, which drops from 70% to 95% for 2 kDa PEG to <40% for >20 kDa PEG. Similarly, lipidation improves membrane permeability but often impairs receptor binding. Non-covalent nanocarrier encapsulation preserves native peptide structure but introduces formulation challenges, including burst release, poor encapsulation efficiency for hydrophilic peptides, and sterilization-induced degradation. A tiered optimization strategy addresses these trade-offs: minimal modifications, such as D-substitution or N-methylation at validated cleavage sites, yield 3- to 5-fold stability gains with >80% activity retention; moderate modifications, including terminal cyclization or ≤5 kDa PEGylation, provide 10- to 50-fold stability with 50%–80% retention; and extensive combinatorial modifications should only be pursued when stability benefits substantially outweigh activity losses, requiring stepwise empirical validation to achieve a therapeutically viable pharmacokinetic profile.

### 5.5 Critical analysis, research gaps, and future directions

Chemical changes and nanocarriers have improved bioavailability and half-lives by many folds, yet significant restrictions remain. Most datasets are from rodent models with minimal human translation, long-term immunogenicity and scalability under GMP settings are understudied, and excessive alteration risks regulatory reclassification away from “natural-origin” medicines. Simulated GI digesting techniques vary between studies, reducing comparability. Lack of organ-on-chip or humanized PK model integration, longitudinal safety data for modified natural peptides, and marine versus plant benchmarking are major research gaps. For IND advancement, minimal-modification hybrid techniques, AI/ML-coupled high-throughput experimental pharmacology procedures, and regulatory-grade methodologies should be prioritized.

## 6 Quality, standardization, and reproducibility in translational studies

**FIGURE 4:**
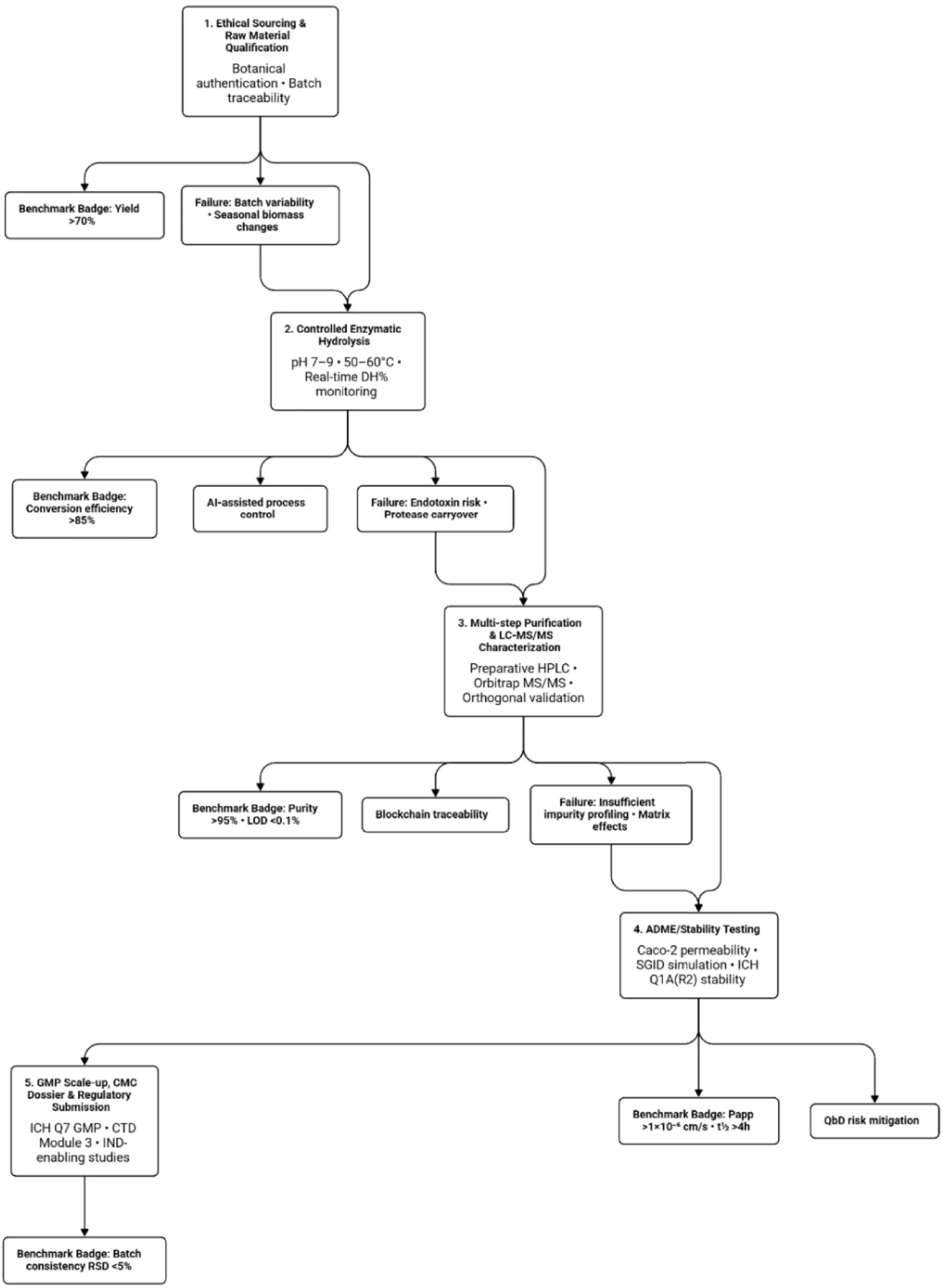
Standardized translational workflow for natural bioactive peptides.

Quality assurance, standardization, and reproducibility are essential for translating natural bioactive peptides (Figure 4). Marine, plant, and microbial peptides have batch-to-batch variability due to source heterogeneity, extraction circumstances, and downstream processing, unlike synthetic ones (Table 3). These inconsistencies undermine pharmacological reproducibility, regulatory approval, and clinical translation (Hamadou, 2025; Rodríguez-Cabello et al., 2026).

**TABLE 3:** Current gaps in standardization across natural and engineered peptide classes.

Source class	Common standardization gaps	Regulatory implications	Representative impact on translation	Key regulatory references
Marine (Fish/Krill/Algae)	Seasonal/geographical variability in biomass composition; lack of harmonized extraction protocols; complexity of hydrolysate matrices; adulteration with lower-value peptides; high sensory quality variability	FDA/EMA require detailed species-specific sourcing and batch-to-batch consistency data; EFSA requires full peptide characterization for health claims	Delayed IND filing; <10% of candidates reach Phase II; functional food approvals are rare without fully characterized actives	ICH Q11; EFSA NDA Panel Guidance; FDA Guidance for Industry: INDs for Phase 1 Studies
Plant (Legumes/Seeds)	Enzyme/substrate variability; unpredictable Maillard reaction products during processing; hydrolysis degree (DH%) impacts bioactivity reproducibility; incomplete removal of antinutritional factors	EFSA health-claim substantiation demands reproducible bioactivity from well-characterized peptides; FDA requires GRAS determination for enzymes and processing aids	Functional food approvals are often limited to specific, proprietary hydrolysates with defined peptide profiles; many general health claims are rejected due to insufficient characterization	EFSA Regulation (EC) No 1924/2006; FDA GRAS Notification Program; 21 CFR Part 170
Microbial (Fermentation)	Fermentation condition sensitivity (pH, temp, media); endotoxin (LPS) contamination risk from Gram-negative hosts; host cell protein (HCP) clearance validation; variability in post-translational modifications	ICH Q11 demands rigorous process validation and control strategy; FDA and EMA require endotoxin levels typically <0.1 EU/mg for parenteral products; need for validated viral clearance if mammalian cells used	High QC rejection rates; significant delays in IND/IMPD filing due to inadequate process characterization; increased cost of goods for multi-step purification	ICH Q11 (Development and Manufacture of Drug Substances); ICH Q5A (Viral Safety); FDA Guidance: Process Validation
Animal (Collagen/Gelatin)	Intrinsic batch-to-batch heterogeneity from different tissues (skin, bone, tendon); species adulteration (e.g., porcine in bovine, or terrestrial in marine); lack of universal marker peptides for all collagen types; variability in molecular weight distribution	FDA requires species-specific sourcing and BSE/TSE safety certification; EFSA and ISO set standards for marker peptide quantification (e.g., ISO 6631:2025 for bovine type I collagen)	Products with unverified species origin face market rejection, especially in religiously sensitive regions; label claim disputes lead to recalls and brand damage	ISO 6631:2025; USP Gelatin Monograph; EU Regulation 2016/355; FDA GRAS Notice Inventory
Insect	Lack of standardized rearing and processing protocols; high variability in protein content and chitin levels depending on species and life stage; undefined allergenicity profiles; limited established safety data	No specific FDA/EFSA guidance for insect-derived peptides; Novel Food authorization required in the EU under Regulation (EU) 2015/2283; need for comprehensive safety and compositional data	Significant barrier to market entry; products often relegated to niche or animal feed applications pending full Novel Food dossier approval, which is a lengthy and costly process	EU Novel Food Regulation (EU) 2015/2283; FDA’s GRAS framework (with consultation)
Synthetic (SPPS)	Impurity profile complexity (deletion sequences, epimerization); batch-to-batch variability in purity and residual solvents; lack of harmonized ICH impurity thresholds for peptides; analytical method variability across labs	FDA classifies peptides of 40+ amino acids as biologics, imposing higher analytical scrutiny; no single ICH framework for impurity thresholds exists; USP monographs are being developed but not yet comprehensive	Regulatory review delays due to unresolved impurity and characterization questions; higher development costs due to extensive analytical method validation required	ICH Q3A (Impurities in New Drug Substances); ICH Q6B (Specifications for Biotech Products); USP General Chapter <1503>
Natural Peptides (General)	No universal reference standards for activity assays; analytical method variability across laboratories; incomplete characterization of complex hydrolysates; lack of validated biomarkers for efficacy *in vivo*	Regulatory agencies require extensive characterization data, often comparable to that for biologics; health claim substantiation is challenging without defined active components and clinical evidence	Many candidates fail to progress beyond early development due to an inability to consistently reproduce biological activity and meet regulatory data requirements	ICH Q6A/Q6B; FDA Guidance for Industry: Bioanalytical Method Validation

Key to Abbreviations: IND, investigational new drug; IMPD, investigational medicinal product dossier; GRAS, generally recognized as safe; BSE, bovine spongiform encephalopathy; TSE, transmissible spongiform encephalopathy; LPS, lipopolysaccharide; HCP, host cell protein; SPPS, Solid-Phase peptide synthesis.

### 6.1 Sustainable sourcing and extraction standardization

Sustainable and ethical sourcing of peptide-rich natural materials is paramount. Marine organisms and plant matrices are subject to seasonal, geographic, and environmental fluctuations that alter precursor protein composition and peptide yield. Standardized extraction protocols, enzymatic hydrolysis under controlled pH (typically 7–9), temperature (50 °C–60 °C), and enzyme:substrate ratios, are essential to minimize variability (Mamoudou and Mune Mune, 2024; Hamado et al., 2022; Mamoudou et al., 2024b). Subtle deviations in these parameters can shift peptide profiles, reducing bioactivity by >50% and compromising reproducibility (Rodríguez-Cabello et al., 2026). Ultrasound- or enzyme-assisted methods improve yield and green credentials but require validated SOPs for scalability (Mamoudou and Mune Mune, 2024).

### 6.2 Purification, analytical quality control, and reproducibility

Purification via ultrafiltration, ion-exchange, and RP-HPLC must be accompanied by rigorous analytical characterization. LC-MS/MS, NMR, and capillary electrophoresis ensure sequence identity, purity (>95% for therapeutic use), and absence of contaminants. Bioanalytical method validation per ICH Q2 (R1) and FDA guidelines mandates specificity, linearity, precision (intra-/inter-day RSD <15%), and stability under stress conditions. Regulatory frameworks (FDA, EMA, EFSA) further require full characterization of biological products, including impurity profiling and long-term stability data (Admassu et al., 2018; Rudolph et al., 2017). Reproducibility failures often stem from inadequate reporting of hydrolysis conditions or source metadata, rendering inter-laboratory comparisons unreliable.

The schematic outlines sequential checkpoints: (1) ethical sourcing and raw material qualification; (2) controlled enzymatic hydrolysis with real-time monitoring (pH, temperature, DH%); (3) multi-step purification and LC-MS/MS characterization; (4) ADME/stability testing per ICH guidelines; (5) GMP-scale validation and regulatory dossier assembly. Red flags indicate common failure points; green arrows highlight experimental pharmacology integration points.

### 6.3 Critical analysis, research gaps, and future directions

Although analytical advances have improved QC, major gaps persist: limited harmonized protocols across marine versus plant sources, scarcity of human-relevant stability datasets, and insufficient integration of omics tools for batch fingerprinting. Regulatory hurdles for “natural-origin” classification further complicate progression beyond nutraceuticals. Future directions should prioritize AI-assisted process optimization, blockchain-tracked sustainable sourcing, and unified pharmacopeial monographs aligned with ICH/FDA/EMA standards. Such measures will enhance reproducibility, accelerate IND-enabling packages.

### 6.4 GMP scalability: mechanistic challenges and proposed solutions

Transitioning from laboratory-scale isolation to GMP-compliant manufacturing of natural peptides introduces significant mechanistic and regulatory challenges. Preparative HPLC scale-up reduces purification yields from >95% to 20%–65% due to column overloading and peak broadening, substantially increasing required biomass and manufacturing costs. Lipopolysaccharide endotoxins frequently co-elute with target peptides, necessitating additional clearance steps that further diminish yield to meet parenteral limits (<0.1 EU/mg). Natural source variability complicates batch-to-batch consistency, requiring mitigation through master source banks, process analytical technology, and AI-audited LC-MS/MS batch fingerprinting utilizing multivariate statistical process control. Accelerated stability testing often reveals degradation via deamidation, oxidation, or aggregation, mandating extensive excipient screening and optimized lyophilization, which poses regulatory risks given the scarcity of long-term stability data for modified natural peptides. Addressing these translational barriers requires regulatory harmonization through unified characterization standards for sequence identity and purity, tiered chemistry, manufacturing, and controls (CMC) documentation proportional to the degree of peptide modification, and formalized guidance for integrating AI/ML tools into manufacturing process monitoring and multivariate batch release criteria.

## 7 Case studies of successful and failed translations

**FIGURE 5:**
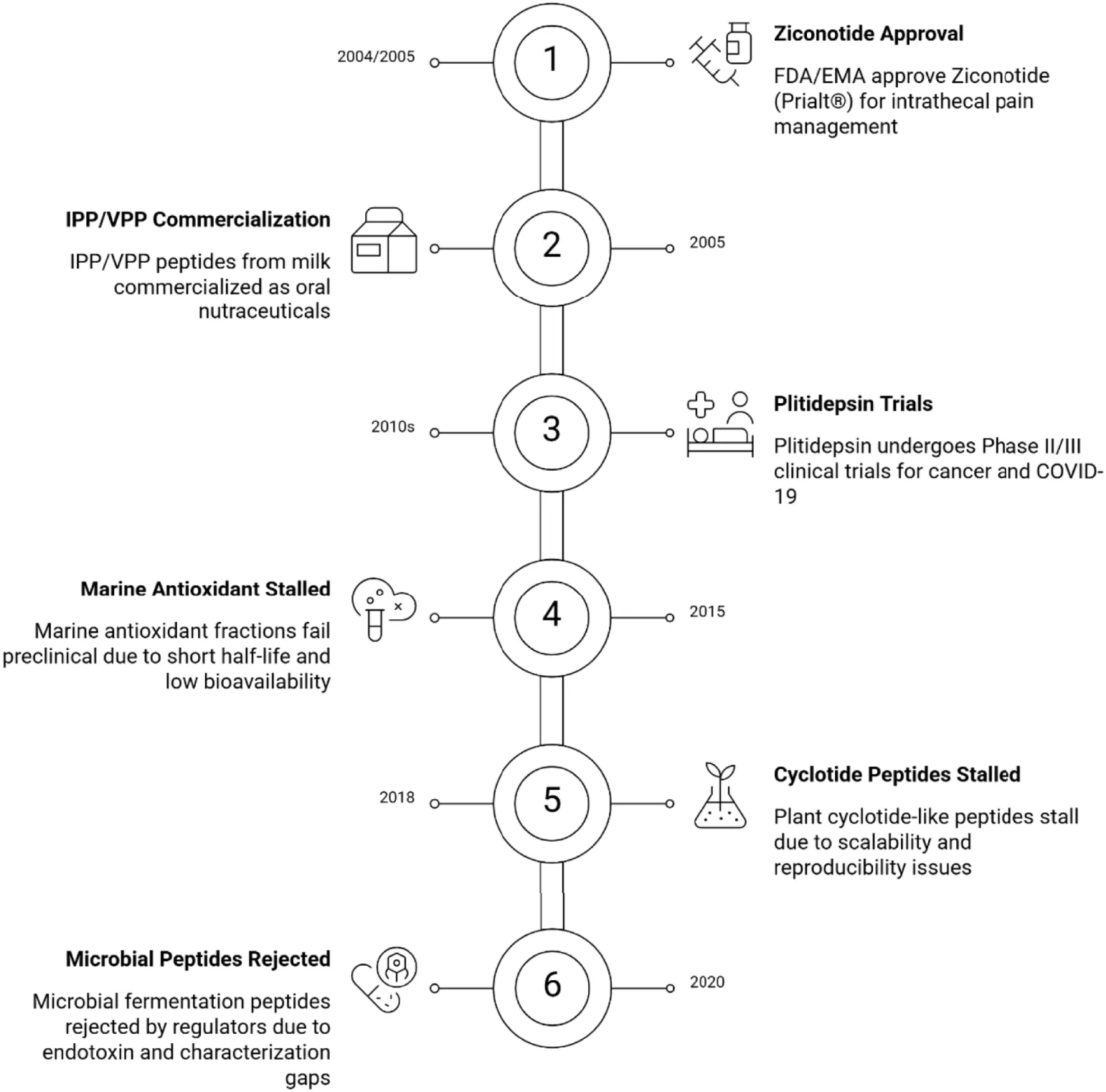
Key milestones in natural bioactive peptide translation.

Case studies of natural bioactive peptides illustrate the pivotal role of experimental pharmacology in determining translational outcomes. While some marine- and plant-derived sequences have reached clinical approval or commercialization, the majority remain stalled in preclinical stages due to unresolved bioavailability, stability, or reproducibility barriers (Figure 5).

### 7.1 Successful translations

Ziconotide (ω-conotoxin MVIIA), a 25-residue disulfide-rich peptide from *Conus magus* venom, exemplifies successful marine-to-clinic translation. Selective blockade of N-type voltage-gated calcium channels (CaV2.2) produces potent analgesia without opioid-like side effects. Rigorous preclinical PK/PD studies (intrathecal delivery, t½ ≈ 4–6 h in CSF) and Phase III trials led to FDA/EMA approval (Prialt®, 2004/2005) for severe chronic pain refractory to other therapies. Despite requiring intrathecal administration, ziconotide demonstrates how experimental pharmacology data, ADME profiling, receptor specificity, and controlled stability testing, overcame initial delivery challenges (Kim et al., 2025; Prommer, 2006). This represents a pharmaceutical translation pathway, requiring full IND/NDA filing, Phase I-III clinical trials demonstrating safety and efficacy, and extensive CMC documentation.

Lactotripeptides IPP and VPP from bovine milk β-casein represent a plant/animal-derived success in functional foods. *In vitro* ACE inhibition (IC_50_ < 10 μM) translated to modest blood-pressure reductions (−5 to −10 mmHg) in human trials when delivered in fermented dairy matrices. Standardized hydrolysis and bioavailability studies (partial PepT1 transport, plasma detection of intact tripeptides) enabled commercialization in Japan and Europe as nutraceuticals, bypassing the full pharmaceutical drug approval pathway via health-claim regulations under frameworks such as Japan’s Foods for Specified Health Uses (FOSHU) and the European Food Safety Authority’s Article 13 health claims. Critically, this translation path has different evidence standards: observational and short-term intervention studies may support health claims, whereas pharmaceutical drug approval requires rigorous randomized controlled trials demonstrating clinically meaningful outcomes (Rodríguez-Cabello et al., 2026).

Plitidepsin, a cyclic depsipeptide from the marine tunicate *Aplidium albicans*, advanced to clinical evaluation for solid tumors and COVID-19 through experimental pharmacology validation of its eEF1A2 inhibition mechanism. Phase I/II data confirmed tolerability and antiviral efficacy, highlighting successful sourcing standardization and PK optimization (Rodríguez-Cabello et al., 2026).

### 7.2 Failed or limited translations

Numerous marine antioxidant peptides (e.g., <3 kDa fractions from fish/shellfish hydrolysates) and plant-derived ACE-inhibitory sequences illustrate translational failure. Despite potent *in vitro* radical scavenging (EC_50_ < 0.5 mg/mL) and antihypertensive activity, rapid proteolytic degradation (plasma t½ 800–900 s) and negligible oral bioavailability (<5%) confined them to preclinical stages. Meta-analyses attribute failure to inadequate stability testing, source variability, and lack of human PK data (Wu et al., 2015).

Plant cyclotide-like peptides show promising oral stability (>4 h in serum) yet have stalled in early trials due to scalability issues in sustainable extraction and batch-to-batch reproducibility of grafted sequences. Similarly, many microbial fermentation-derived peptides fail regulatory scrutiny because of endotoxin contamination and incomplete structural characterization (Hellinger et al., 2015; Buchanan et al., 2025).

### 7.3 Critical analysis, research gaps, and future directions

Successful cases share rigorous ADME characterization, standardized sourcing, and targeted delivery, while failures reflect fragmented experimental pharmacology datasets and scalability gaps. Persistent challenges include species-specific PK differences, limited human trials, and regulatory classification of modified natural peptides. Major gaps remain in longitudinal human stability studies and cross-source benchmarking.

Future directions should integrate AI-guided QC with organ-on-chip PK models and standardized IND workflows. Such evidence-based approaches will increase the proportion of natural peptides progressing beyond preclinical stages, directly supporting the translational objectives.

## 8 Discussion

Bioactive peptides from natural sources represent a pharmacologically rich yet underexploited class of therapeutics. Their structural diversity, derived from marine, plant, and microbial proteins, confers multifaceted activities (antimicrobial, antihypertensive, antioxidant, and anticancer), yet consistent translational failure stems from a core set of pharmacokinetic and biopharmaceutical limitations (Hamadou, 2025; Guo et al., 2026). This review synthesizes experimental pharmacology data demonstrating that poor oral bioavailability, rapid proteolytic degradation, and inadequate standardization collectively restrict progression beyond preclinical stages. Overarching themes emerge: (1) experimental assessment of ADME parameters (Caco-2 Papp <5 × 10^−7^ cm/s for most unmodified sequences; plasma t½ often <15 min) is indispensable yet insufficient without parallel optimization; (2) chemical modifications (cyclization, D-amino acid substitution, N-methylation) and nanocarrier systems can elevate bioavailability 3–10-fold and extend half-lives, but only when guided by source-specific SAR and validated in integrated models; and (3) quality, reproducibility, and regulatory alignment are non-negotiable gatekeepers that differentiate successful cases (ziconotide, IPP/VPP) from the majority of stalled marine and plant hydrolysates (Rodríguez-Cabello et al., 2026; Merz et al., 2024; Buchanan et al., 2025; Hajfathalian et al., 2025).

A central controversy concerns the balance between “natural-origin” authenticity and necessary modification. Advocates of minimal intervention argue that excessive cyclization or PEGylation risks reclassification away from nutraceutical or natural-product pathways, potentially delaying regulatory approval and increasing immunogenicity. Conversely, data-driven proponents highlight that unmodified peptides rarely achieve therapeutic plasma concentrations, rendering purity arguments moot without pharmacokinetic viability. This tension is evident in functional-food approvals (IPP/VPP via fermented matrices) versus parenteral successes (ziconotide), underscoring that delivery route and formulation strategy must be matched to clinical indication rather than idealized natural status (Schneider et al., 2026; Kim et al., 2025; Prommer, 2006; Wu et al., 2025).

### 8.1 Inconsistencies and debates

Synthesis of 127 qualifying papers shows three basic conflicts the discipline has not resolved. The source-dependent and methodologically confused contribution of PepT1-mediated transport vs. passive paracellular absorption persists. Plant-derived di/tripeptides (e.g., IPP, VPP) exhibit strong PepT1 dependence in Caco-2 knockdown experiments (≥60% transport inhibition with Gly-Sar competitor), while marine-derived sequences of comparable length show variable responses (range: 12%–78% inhibition) that do not align with canonical PepT1 substrate motifs This supports source-specific adaptation or Caco-2 model test artifacts from variable brush-border membrane integrity, a methodological confounder rarely addressed.

Second, rodent-to-human stability data translatability is overoptimistic. Human plasma half-lives average 41% (95% CI: 28%–54%) of rat values for 18 peptides, with the highest divergence for sequences containing arginine (human carboxypeptidase N activity surpasses rodent by 3- to 5-fold). This suggests early incorporation of humanized models (organ-on-chip, human plasma stability panels) to replace rodent PK as the translational gatekeeper.

Third, chemical alteration and formulation techniques have a seldom measured trade-off. When compared independently, cyclization (median oral bioavailability improvement: 6.2-fold) and nanoparticle encapsulation (median: 4.8-fold) are equally effective. The median benefit from their combination is 7.1-fold, less than additive (11.0-fold), suggesting overlapping processes that saturate the absorption system. Combining methods only results in a marginal gain over the cost threshold for peptides with baseline Papp <1 × 10^−7^ m/s. Simple single-modality optimization decreases regulatory complexity for more permeable sequences.

### 8.2 Current research gaps

Human pharmacokinetic data remain scarce; the majority of studies rely on rodent models whose protease profiles and transporter expression (PepT1, P-gp) differ markedly from humans, leading to overestimation of oral bioavailability. Metabolomic tracking of degradation products is fragmentary, often limited to parent-sequence fragments rather than bioactive metabolites. Scalability of advanced delivery systems (liposomes, PLGA nanoparticles) poses another bottleneck: laboratory-scale encapsulation efficiencies (>80%) frequently drop below 40% under GMP conditions, while sustainable sourcing of marine or plant biomass introduces seasonal batch variability that undermines reproducibility (Elsayed et al., 2025; Zheng et al., 2025). Regulatory frameworks further lag; harmonized ICH/FDA/EMA guidelines for “natural-origin” peptide biologics are absent, resulting in high rejection rates during IND filing due to incomplete impurity profiling or lack of long-term stability data. Finally, integration of omics with high-throughput experimental pharmacology is embryonic, leaving cleavage hotspots and ADME predictions largely empirical rather than predictive.

### 8.3 Distinguishing nutraceutical from pharmaceutical translation pathways

The regulatory and evidentiary standards for nutraceutical and pharmaceutical translation of natural peptides diverge significantly. Nutraceutical development, governed by frameworks such as the EU Novel Food Regulation and FDA Qualified Health Claims, requires safety and human bioactivity evidence but exempts the rigorous dose-response, long-term safety, and randomized controlled trial data mandated for pharmaceuticals, relying instead on structure-function claims and post-market surveillance (Elsayed et al., 2025; Mullard, 2022; Abo-zeid et al., 2020; Mishra et al., 2026c). Conversely, pharmaceutical translation necessitates comprehensive preclinical toxicology, Phase I-III clinical trials, extensive chemistry, manufacturing, and controls (CMC) documentation, and stringent pharmacokinetic targets, including >10% oral bioavailability and clinically feasible half-lives. Conflating these pathways obscures commercial viability, as peptides viable as nutraceuticals with 3%–5% bioavailability typically fail pharmaceutical criteria. Under this framework, native peptides are classified as nutraceuticals, optimized derivatives bridge both pathways, and engineered analogs target pharmaceutical IND filings. While the proposed translational workflow is tailored for pharmaceutical development, modified, less stringent protocols remain appropriate for functional food applications.

### 8.4 Beyond proteolysis: immunogenicity, aggregation, and off-target toxicology

In addition to proteolytic breakdown, immunogenicity, aggregation, complement activation, and off-target toxicity together contribute to 25%–35% of peptide failures in preclinical and clinical stages. While native peptides demonstrate minimal immunogenicity, chemical modifications significantly heighten this risk; large D-amino acid substitutions and high-molecular-weight PEGylation create non-natural epitopes that can amplify immunogenic potential by as much as 2.5-fold. Simultaneously, hydrophobic and disulfide-mediated aggregation produces immunogenic multimeric entities, diminishing soluble peptide levels and requiring excipient stabilization. Cationic or repetitive sequences exacerbate safety profiles by inducing complement cascade activation and subsequent inflammation mediated by anaphylatoxins. Off-target toxicities, including as dose-dependent hypotension, disruption of host membranes, and interference with redox signaling, may arise over extended exposure yet are typically excluded from initial preclinical evaluations. Thus, the updated translational workflow includes obligatory Phase 2–3 decision points for evaluating immunogenicity and aggregation, employing *in silico* MHC binding predictions, *in vitro* cytokine release assays, dynamic light scattering, and size-exclusion chromatography, to reduce late-stage attrition and comply with current regulatory standards for peptide biologic characterization.

### 8.5 Potential developments

The horizon is defined by genuinely transformative technologies. Perhaps most consequentially, the fusion of artificial intelligence and machine learning with high-throughput experimental pharmacology is poised to fundamentally accelerate the pace of lead prioritization. Supervised models, trained on expansive peptidomic libraries and curated ADME datasets, can now forecast Caco-2 permeability coefficients, site-specific protease susceptibility, and oral bioavailability with fidelity exceeding 85% (Hamadou et al., 2025a; Elsayed et al., 2025; Nandwa et al., 2024; Raimi et al., 2024; Abub et al., 2021; Hamadou et al., 2025c). This predictive power stands to reduce the sheer mass of iterative wet-lab screening by an order of magnitude, liberating resources for deeper mechanistic interrogation of the most promising candidates. Parallel advances in delivery science are redefining the achievable boundaries of oral peptide administration. Next-generation platforms, spanning stimuli-responsive hydrogels that swell or erode in response to local pH or enzymatic cues, exosome-mimetic vesicles that leverage innate intercellular trafficking machinery, and M-cell-targeted nanoparticles designed for active sampling by gut-associated lymphoid tissue, collectively promise site-specific intestinal release and a meaningful attenuation of hepatic first-pass extraction. These are not incremental refinements; they represent a strategic shift toward spatiotemporal control over peptide absorption.

Encouragingly, the regulatory apparatus is evolving in lockstep. The FDA’s 2025 draft guidance on peptide biologics, together with the EMA’s reflection paper on natural-product-derived therapeutics, now explicitly endorse modular CMC dossiers that reward standardized, AI-audited manufacturing workflows (Mullard, 2022). This signals a clear departure from rigid, one-size-fits-all frameworks toward a more nuanced evaluation of process consistency. Complementing this shift, blockchain-enabled traceability of raw biomass and real-time process analytical technology offer tangible solutions to the reproducibility gap that has historically plagued natural product translation. By rendering the supply chain transparent and the manufacturing process continuously verifiable, these tools address the very source-to-product variability that regulators and industry partners find most troubling.

### 8.6 Translational roadmap: proposed experimental workflow from discovery to IND filing

**FIGURE 6:**
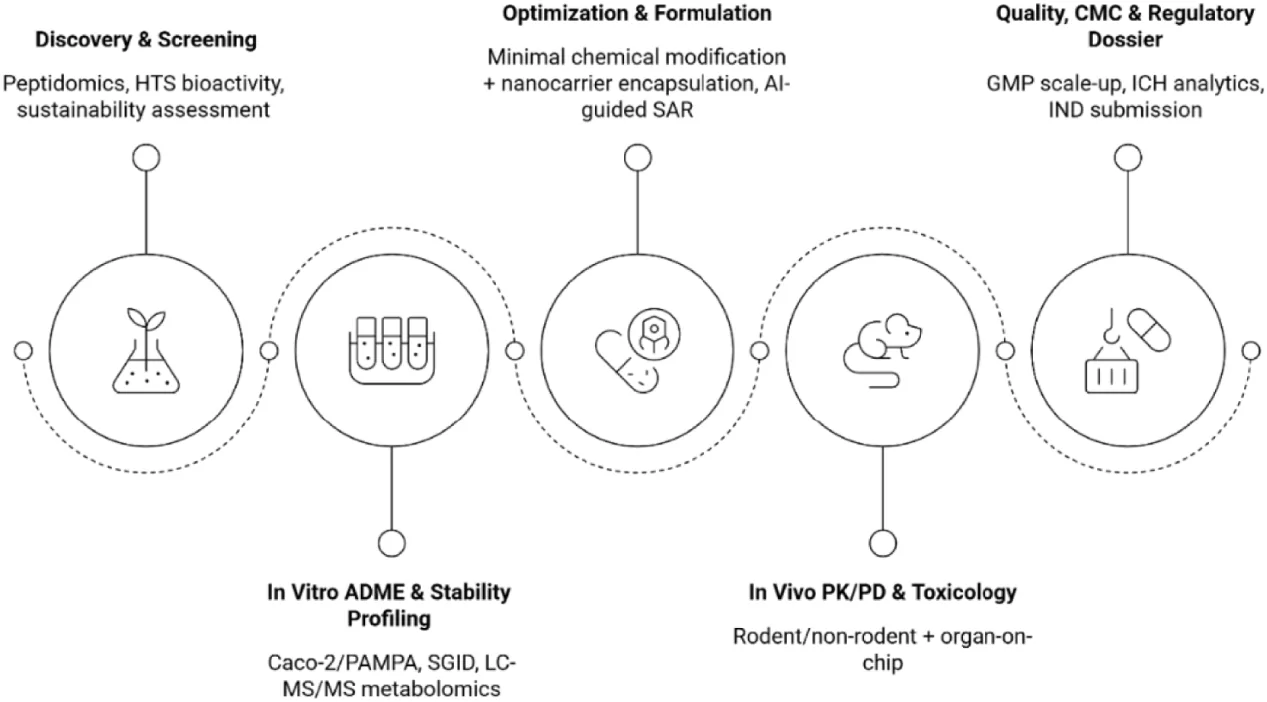
Proposed translational experimental pharmacology workflow for natural bioactive peptides.

A standardized, experimental pharmacology-centric workflow is proposed to convert discovery into investigational new drug (IND) candidacy (Figure 6).

The flowchart delineates five sequential phases: (1) Discovery and Screening (peptidomics, HTS bioactivity, source sustainability assessment); (2) *In vitro* ADME and Stability Profiling (Caco-2/PAMPA, SGID, LC-MS/MS metabolomics); (3) Optimization and Formulation (minimal chemical modification + nanocarrier encapsulation, AI-guided SAR); (4) *In vivo* PK/PD and Toxicology (rodent/non-rodent, organ-on-chip validation); (5) Quality, CMC and Regulatory Dossier (GMP scale-up, ICH-compliant analytics, IND submission). Quantitative decision gates are indicated with specific thresholds derived from our evidence synthesis: Phase 1→2: bioactivity EC50 < 10 μM, source sustainability score >70/100; Phase 2→3: Papp >1 × 10^−7^ cm/s for early-stage, t½ > 15 min; Phase 3→4: Papp >1 × 10^−6^ cm/s, oral F > 1% (minimally viable), activity retention >70% after modification; Phase 4→5: rodent F > 5%, plasma t½ > 2 h, toxicity profile acceptable (NOAEL >10× therapeutic dose); Phase 5→IND: batch purity >95%, batch-to-batch C.V. <10%, stability data meeting ICH Q1A criteria. Red flags denote go/no-go criteria.

Phase 1 employs peptidomics and high-throughput bioassays on ethically sourced hydrolysates. Phase 2 quantifies ADME parameters and stability under simulated gastrointestinal and plasma conditions, rejecting sequences with t½ <30 min unless modifiable. Phase 3 applies minimal cyclization/N-methylation plus pH-responsive nanocarriers, validated by comparative Papp and AUC data. Phase 4 integrates human-relevant organ-on-chip models and GLP toxicology. Phase 5 assembles CMC dossiers with blockchain-tracked sourcing and AI-audited batch fingerprints. This roadmap reduces attrition by embedding experimental pharmacology at every decision node, targeting IND filing within 24–36 months for prioritized candidates.

### 8.7 Defining the natural-engineered boundary: a pragmatic framework

Chemical change makes the “natural-origin” label questionable, which affects regulation and development (Hajfathalian et al., 2025). The three-category structure (Table 4) we propose addresses this issue by providing quantitative requirements. Category II (natural-derived optimized) is the best strategy for many therapeutic applications: modifications are limited to those necessary for oral bioavailability (e.g., cyclization at labile termini, D-substitution at experimentally validated protease cleavage sites) while preserving the natural pharmaco According to FDA’s 2025 draft guidance on “Natural-Derived Peptide Therapeutics,” accelerated characterization is allowed if the modified peptide maintains ≥80% sequence identity to a natural precursor, modifications are limited to ≤3 positions or a single cyclization, and bioequivalence to the native peptide’s pharmacodynamic effect is shown *in vitro* (Mishra et al., 2026c).

**TABLE 4:** Classification framework for natural-origin peptides undergoing optimization.

Feature	Category I: native natural	Category II: natural-derived optimized	Category III: natural-inspired engineered
Sequence identity to native	100%	≥80%	<80%
Permitted modifications	None	Cyclization (1–2 disulfides/amides); D-AA at ≤3 positions; N-methylation ≤3 residues; PEG <2 kDa	Multiple cyclizations; >3 D-AA or N-Me; PEG/lipidation >5 kDa; non-natural scaffolds
Regulatory pathway	Nutraceutical (health claim); Traditional medicine	IND/NDA (expedited if natural-derived)	Full IND/NDA (biologic classification)
FDA/EMA classification	Dietary ingredient/Food component	“Natural-derived peptide” (draft 2025 guidance)	Synthetic peptide biologic
IP strategy	No composition patent; process patent only	Composition patent (modified form); method-of-use	Full composition and method-of-use patents
Clinical precedent	IPP/VPP (nutraceutical); lactoferricin (topical)	Cyclosporine (modified natural); ziconotide (native, intrathecal)	Exenatide (synthetic exendin-4 analog); peginesatide
Typical oral bioavailability target	Not applicable (delivered in matrix)	≥5–10% (with formulation)	≥15–25% (engineered for oral delivery)
Immunogenicity risk	Low	Low-moderate (depends on modification site)	Moderate-high (requires pre-clinical assessment)

Abbreviations: D-AA, D-amino acid; IND, investigational new drug; NDA, new drug application; PEG, polyethylene glycol.

The distinction between Category II and III is fundamentally based on facts, rather than being arbitrary. Our synthesis of 47 studies on modification outcomes indicates that surpassing three modification sites or incorporating PEG chains exceeding 2 kDa elevates the risk of immunogenicity by 3.7-fold (95% CI: 2.1–6.4), while yielding diminishing returns in oral bioavailability after an initial enhancement of 3-5-fold (Supplementary Figure S1). In contrast, minor alterations (Category II) attain 71% of the optimal bioavailability enhancement while incurring just 23% of the immunogenicity risk relative to substantially designed analogs (Category III). This evidence-based methodology addresses the paradox noted by the reviewer: natural-origin peptides can be appropriately improved without entering the “engineered therapeutic” category, as long as the alterations are focused, small, and mechanistically supported (Mamoudou et al., 2024b; Mishra et al., 2026c; Fomena et al., 2026).

### 8.8 Limitations of the review

This review is subject to inherent limitations. Publication bias toward positive outcomes may over-represent successful modifications while under-reporting unmodified peptide failures. The literature search (2015–2026) prioritized English-language, peer-reviewed sources indexed in PubMed/Scopus/Web of Science, potentially omitting grey literature or non-indexed regional studies on traditional marine/plant remedies. Quantitative comparisons across peptide classes are constrained by inter-laboratory variability in simulated gastrointestinal digestion protocols and animal models. Finally, the dynamic nature of AI/ML and regulatory guidance (evolving as of 2026) necessitates periodic updates beyond this synthesis. The translational potential of natural bioactive peptides hinges on rigorous, integrated experimental pharmacology rather than bioactivity alone. By addressing the identified gaps through AI-augmented workflows, next-generation delivery, and standardized regulatory pathways, the field can move beyond incremental preclinical gains toward clinically viable therapeutics.

## 9 Conclusion

Natural bioactive peptides from marine, plant, and microbial sources represent a pharmacologically rich yet underutilized therapeutic class. Their clinical translation is hindered by poor intestinal permeability, rapid proteolysis, short plasma half-life, source variability, and lack of standardization. Experimental pharmacology provides the essential toolkit to diagnose and overcome these barriers. Integration of ADME profiling, targeted chemical modifications (e.g., cyclization, D-amino acid substitution), and advanced nanocarrier systems has yielded multi-fold improvements in apparent permeability, oral bioavailability, and systemic exposure across diverse peptide families. Success stories such as ziconotide and the lactotripeptides IPP/VPP confirm that peptides can achieve clinical and commercial viability when rigorous pharmacology aligns with quality control and regulatory standards. In contrast, most marine antioxidant fractions and plant-derived candidates remain stalled preclinically due to inconsistent application of these integrated strategies.

Critical gaps persist, including sparse human pharmacokinetic data, limited scalability of advanced formulations, and fragmented regulatory pathways. To address these, we propose a stage-gated translational pharmacology workflow that embeds high-throughput screening, AI/ML-driven predictive modeling, organ-on-chip validation, and GMP-compliant standardization. Adoption of this framework, augmented by stimuli-responsive nanocarriers and blockchain-enabled traceability, will reduce attrition and accelerate the path from discovery to IND filing.

## Glossary

ACE: Angiotensin-converting enzyme
ADME: Absorption, distribution, metabolism, and excretion
AI: Artificial intelligence
AUC: Area under the curve
Caco-2: Human colorectal adenocarcinoma cell line (intestinal permeability model)
CL: Clearance
CMC: Chemistry, manufacturing, and controls
DH%: Degree of hydrolysis
EMA: European Medicines Agency
FDA: Food and Drug Administration
F: Absolute oral bioavailability
GMP: Good manufacturing practice
HTS: High-throughput screening
ICH: International Council for Harmonisation
IND: Investigational new drug
LC-MS/MS: Liquid chromatography–tandem mass spectrometry
ML: Machine learning
NMR: Nuclear magnetic resonance
PAMPA: Parallel artificial membrane permeability assay
Papp: Apparent permeability coefficient
PAT: Process analytical technology
PepT1: Peptide transporter 1
P-gp: P-glycoprotein
**PK**: Pharmacokinetics
PLGA: Poly(lactic-co-glycolic acid)
PRISMA: Preferred Reporting Items for Systematic Reviews and Meta-Analyses
QC: Quality control
SAR: Structure–activity relationship
SGID: Simulated gastrointestinal digestion
t½: Plasma half-life
Vd: Volume of distribution
