# Supplementary material for: From bench to bedside: experimental pharmacology approaches to overcome bioavailability, stability, and translational barriers of bioactive peptides from natural sources
Source: Front Pharmacol. 2026 Aug 12;17:1863016. doi: 10.3389/fphar.2026.1863016 (PMC13507803; doi:10.3389/fphar.2026.1863016)
Supplement: Supplementary file 1 [file Image1.pdf]

Supplementary figure

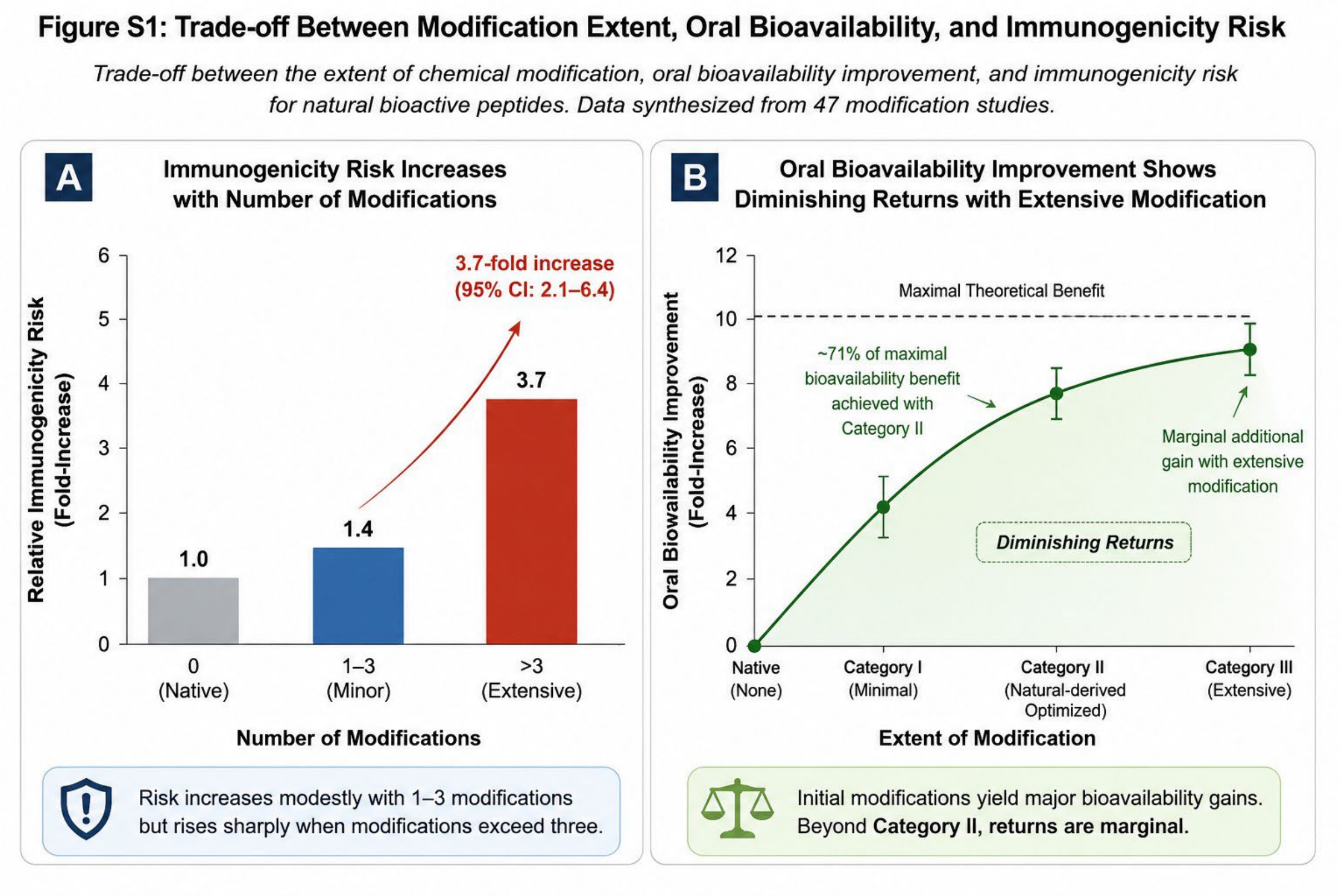

Figure S1. Trade-off between modification extent, oral bioavailability, and immunogenicity risk
