# Supplementary material for: From bench to bedside: experimental pharmacology approaches to overcome bioavailability, stability, and translational barriers of bioactive peptides from natural sources
Source: Front Pharmacol. 2026 Aug 12;17:1863016. doi: 10.3389/fphar.2026.1863016 (PMC13507803; doi:10.3389/fphar.2026.1863016)
Supplement: Supplementary file 2 [file Table1.docx]

**Supplementary data**

**Table S1: Permeability Coefficients of Unmodified Natural Bioactive Peptides**

| **Peptide Sequence** | **Source Class** | **Papp (×10⁻⁷ cm/s)** | **Reference / Note** |
| --- | --- | --- | --- |
| Aggregated Data (n=47) | Marine, Plant, Animal | Median: 4.7 (IQR: 2.1 – 8.3) | Systematic compilation from 47 distinct peptide sequences. |
| Marine-Derived Peptides (Median) | Marine | Median: 2.3 | Consistently lower permeability compared to plant-derived peptides. |
| Plant-Derived Di/Tripeptides (Median) | Plant | Median: 6.8 | Higher median permeability within the plant source category. |
| WDHHAPQLR (RAP) | Plant (Rapeseed) | 8.2 | Example of a plant-derived peptide with relatively high permeability. |
| VERG | Marine (Krill) | 2.3 | Example of a marine-derived peptide with lower permeability. |

***Note:*** *This table is a reconstruction based on the systematic compilation mentioned in the main manuscript. The specific peptide sequences and their individual Papp values were not listed in the main text; therefore, the table below is populated with the aggregated data and illustrative examples provided in your review.*
